# Femoral neck fracture patients with ischaemic stroke choose hemiarthroplasty or constrained liner total hip arthroplasty? A retrospective comparative study of 199 cases

**DOI:** 10.3389/fsurg.2023.1258675

**Published:** 2023-10-16

**Authors:** Jia Huo, Sikai Liu, Mengnan Li, Zeming Liu, Xuzhuang Ding, Bo Liu, Huijie Li, Yongtai Han

**Affiliations:** Department of Orthopaedic Surgery, the Third Hospital of Hebei Medical University, Shijiazhuang, China

**Keywords:** femoral neck fracture, dislocation, ischaemic stroke, total hip arthroplasty, hemiarthroplasty, constrained liner

## Abstract

**Background:**

The objective of this study was to assess the long-term survival rate, complications, as well as the clinical and radiological outcomes of hemiarthroplasty and total hip arthroplasty using constrained polyethylene liners in patients with ischemic stroke.

**Methods:**

This study was a retrospective cohort study that included patients with ischemic stroke who underwent hip arthroplasty from March 2010 to September 2017. In the Constrained Acetabular Liners (CAL) group, patients received an uncemented acetabular shell with a constrained polyethylene liner. The Dual Mobility (DM) group underwent hemiarthroplasty (HA). Additionally, hip function, range of motion, quality of life, the incidence of clinical complications, and prosthesis stability were investigated.

**Results:**

96 patients with unilateral femoral neck fractures who underwent hip replacement with CAL were included in the CAL group, while 103 patients who underwent hip replacement with a dual mobility head were included in the DM group. VAS, and SF-36 data were available for both CAL and DM groups. At the 1-year postoperative follow-up, the HHS in the CAL group was significantly lower than that in the DM group (80.83 ± 3.91 vs. 83.17 ± 4.15, *P* < 0.05). The VAS score in the CAL group peaked at the 1-year follow-up (2.07 ± 0.91 vs. 1.49 ± 0.85, *P* < 0.05). However, there were no significant differences between the two groups in terms of HSS, VAS, and SF-36 at the last follow-up after surgery. Operative time and the amount of bleeding in the DM group were significantly lower than those in the CAL group (105.30 ± 29.68 vs. 94.85 ± 31.07; 355.11 ± 123.95 vs. 302.22 ± 107.68, *P* < 0.05). Additionally, there was no significant difference in the mean leg length discrepancy between the two groups.

**Conclusion:**

The clinical, imaging, and postoperative complications of the CAL and DM groups were analyzed. The prognosis for DM appears to be more beneficial for early patient recovery, but a higher likelihood of recurrent dislocation is observed. CAL offers excellent stability for primary THA in high-risk patients; however, attention should be given to preventing aseptic loosening.

## Introduction

Dislocation is one of the common complications following primary total hip arthroplasty (THA) in patients with femoral neck fractures (1). Previous studies have indicated that the dislocation rate of the hip joint during initial THA ranges from 0.5% to 3% and increases to 11% during total hip revision surgery (1). Therefore, preventing postoperative dislocation remains an important issue in clinical practice (2). However, risk factors related to patients, surgeons, and implants have been identified previously (3). Several advancements have been made in surgical techniques, implant placement, and implant materials to minimize postoperative instability. However, despite these improvements, the occurrence of instability remains a significant issue.

A previous study has identified numerous risk factors for recurrent dislocation, including prosthesis malposition, abductor muscle weakness, surgical approach, surgeon volume, implant design, and proximal bone loss (4, 5). While some studies (6–9) suggest that adopting specific surgical approaches may be associated with a lower dislocation rate, this effect may be more closely tied to the skill level of the surgeon rather than differences in the approach itself. The variations in dislocation rates between Direct Anterior Approach (DAA) and Posterior Approach (PA) or Lateral Approach (LA), as mentioned in those studies, are partially due to the learning curve surgeons undergo when adopting new techniques or approaches. During the initial stages of this learning curve, surgeons may encounter technical challenges that could influence surgical outcomes, including the incidence of complications. However, as the surgeon's proficiency improves, these differences may gradually diminish until reaching a similar level (10). In our study, the surgical method chosen by our surgeons was the Posterior Approach, and they had already overcome the learning curve, possessing extensive experience with this approach. In this context, the surgeon's skill proficiency becomes a pivotal factor in ensuring surgical success and reducing the risk of complications. Therefore, the findings of this study also underscore the significant role of the surgeon's expertise in achieving surgical success. In conclusion, while different surgical approaches may be associated with complications such as dislocation in specific scenarios, the experience and proficiency of the surgeon remain equally critical factors. Through comprehensive learning and practice, surgeons can better manage risks, ensuring the safety and success of surgeries. When making decisions about surgical approaches and selecting surgeons, the specific circumstances of the patient and the professional capabilities of the medical team should be taken into account.

Elderly patients with strokes often experience reduced muscle strength and weakness, which can lead to decreased physical activity. Abductor muscle weakness, in particular, is a critical factor contributing to dislocation.

To prevent recurrent dislocations resulting from instability, adjustments to prosthesis positioning, selection of different ball head sizes, and the choice between dual mobility (DM) and constrained acetabular liners (CAL) are possible strategies. Recent reports have demonstrated that using CAL can significantly reduce the risk of recurrent dislocations due to instability, but the failure rate remains uncertain (11–13). There is limited research on whether using CAL in elderly stroke patients might increase the incidence of postoperative complications, potentially affecting limb function. The objectives of this study were to investigate the differences between using CAL and traditional prostheses in elderly stroke patients, including postoperative complications, prosthesis survival rates, clinical and imaging evaluations, and to determine appropriate treatment strategies for these patients.

## Method

This study was conducted at the hospital from March 2010 to September 2017 and received approval from our ethics committee.

The inclusion criteria for patients are as follows: (1) The history of ischaemic stroke more than 1 year without other surgical contraindication; (2) The quadriceps muscle strength of the affected limb is level 3 or 4; (3) The displacement of femoral neck fractures is evident, making reduction challenging, and unilateral femoral neck fractures occurring within <7 days; (4) The ability to provide informed consent. The exclusion criteria for patients are as follows: (1) Patients with severe cardiopulmonary dysfunction cannot tolerate the surgery; (2) Primary or metastatic malignant tumor or other pathological fracture of the femoral neck; (3) Previously underwent hip joint surgery for other reasons; (4) Tuberculosis or suppurative joint of the hip joint.

This study is a retrospective research project, and the choice of implants is primarily based on the surgical preferences of the operating physician at that time, as well as the patients' preferences.

The same group of surgeons performs all surgical procedures. Prophylactic antibiotics were used half an hour before surgery.

### Surgical procedure in CAL group

The Constrained Liner System (Chun Li Co, Bejing, China) was used in CAL group. Firstly, after successful anesthesia, the patient should be placed in a lateral position, undergo routine disinfection, and lay a sterile towel. Both groups of patients underwent the posterior lateral approach. Secondly, incision of skin and subcutaneous tissue, expose the external rotator muscle around the hip joint and cut it off to expose and open the joint capsule. Surgical procedure in CAL group: After dislocating the hip joint, perform osteotomy at 1–1.5 cm above the lesser trochanter to the junction of the lateral end of the femoral neck and the greater trochanter. Then, the acetabulum explosion was performed. Grind off the acetabular cartilage and some subcortical bones, while retaining some subchondral and cancellous bones. Then the acetabular component and CAL with suitable size were implanted. Next, induce and rotate the hip to expose the femoral neck osteotomy surface and the compressor was used for metaphyseal medullary preparation. Then, the anatomical prosthesis and ceramic head were implanted, and the joint reduction was performed.

### Surgical procedure in DM group

After remove the femoral head, perform osteotomy at 1–1.5 cm above the lesser trochanter to the junction of the lateral end of the femoral neck and the greater trochanter. Then the metaphyseal medullary preparation was performed, and after that, the anatomical prosthesis and dual mobility head were implemented.

While some studies suggest that the DAA has a lower dislocation rate compared to posterior or lateral approaches, this observation is often made when surgeons are still on their learning curve. In Chen X ’s study, which compared patients undergoing DAA and PA, there was no significant difference in dislocation rates. Early dislocation in these patients was primarily attributed to the surgeon's lack of mastery and being within the learning curve. Once surgeons become proficient in the DAA or PA approaches, they report significantly lower dislocation rates, leading to roughly equivalent dislocation rates between the two surgical methods. In this study, all patients underwent the posterior lateral approach, and the surgeons had substantial experience with this approach, having already passed the learning curve (6–10).

Method for checking the quadriceps muscle strength: The patient is in a supine position, with lower leg sagging outside the bed edge, extending the lower leg and exerting force to resist. Level 3: The limbs can be lifted off the bed surface. Level 4: Able to move under light resistance.

After surgery, all patients received intravenous antibiotics to prevent infection, and those without contraindications for anticoagulants were administered anticoagulant treatment for five weeks to prevent deep vein thrombosis. At 24 h after operation, the load was allowed to the patients with crutches.

Experienced clinical physicians assessed relevant prognostic indicators before surgery, at 1-year post-surgery, and during the last follow-up. These indicators included the Harris Hip Joint Score (HHS), Visual Analogue Scale (VAS), and Short Form-36 Health Survey score (SF-36; www.sf-36.org). HHS was assessed only at 1 year after surgery and during the last follow-up period. HHS is a common prognostic tool after THA (14) and is also used to evaluate femoral neck fractures (15). The HHS consists of 10 items, with a score of <70 points considered inferior, 70–79 as tolerable, 80–89 as good, and 90–100 as excellent. VAS measures pain on a scale of 0 to 10, where 0 indicates no pain, 10 indicates severe pain, and the middle range represents varying degrees of pain. SF-36 scores encompass Physical Function (PF), Role Physical (RP), general health self-assessment (GH), social function (SF), bodily pain (BP), Vitality (VT), Mental Health (MH), and role Emotional (RE) (16). Operation time data was collected from operative records, ranging from skin incision to surgical closure. The amount of bleeding in this study refers to intraoperative blood loss (17).

At the last postoperative follow-up, hip flexion and extension range of motion (ROM) were visually estimated by one of the senior orthopedic surgeons. Anteroposterior and lateral x-ray images of the pelvis and hip were taken to assess postoperative leg length discrepancy. The follow-up time for radiology: 7 days, 1 month, 3 months, 6 months, 1 year after surgery, and once a year thereafter. The non-weight-bearing was performed to calculate limb length discrepancy. The following landmarks are identified on a pelvic radiograph: the centers of the lesser trochanters at the femurs and radiological teardrops. Measuring the distance separating these two landmarks after operation (18). Stress shielding was classified according to Engh's classifications, ranging from 0 (none) to 4 (4th degree) (19).

The postoperative complication of this study included periprosthetic femoral fracture, dislocation, aseptic loosening, and infect.

Statistical tests were performed using SPSS version 19 (SPSS Inc, Chicago, IL). The results are presented as the mean ± standard deviation. The Chi-square test was used to analyze differences such as complications and limb-length discrepancies between the 2 groups. The Mann–Whitney *U*-test was used to analyze compare the HHS, VAS, the offset, the Engh score, and the SF-36 between 2 groups. *P* < 0.05 was considered statistically significant.

## Result

### Demographic information

96 patients with unilateral femoral neck fractures who underwent hip replacement with CAL were included in the CAL group. 103 patients who underwent hip replacement with dual mobility head were included in the DM group. Their baseline characteristics are listed in Table 1.

**Table 1:** Demographic information.

Patients’ characteristics	CAL group (*n* = 96)	DM group (*n* = 103)	*Z*	*P*
Gender				
Male	34	40	0.249^b^	0.661
Female	62	63		
Age (years)	60.43 ± 7.54	58.40 ± 6.49	−1.635^a^	0.102
BMI (kg/m^2^)	24.62 ± 3.73	24.80 ± 4.02	0.908^a^	0.364
Surgical side				
Left	46	44	0.524^b^	0.479
Right	50	59		
Muscle strength of the affected lower limb				
Level 3	44	50	0.146^b^	0.777
Level 4	52	53		
Follow up time (Months)	69.48 ± 6.62	70.62 ± 6.06	1.597	0.110

^a^ Mann–Whitney *U*-test.

^b^ Chi-square test.

Regarding demographic factors, no significant difference in age, sex, body mass index, or muscle strength was retrieved (Table 1).

### Clinical outcomes

For 96 patients in the CAL group and 103 patients in the DM group, HHS, VAS, and SF-36 scores were assessed before surgery, at 1 year postoperatively, and at the last follow-up (Table 2). At the 1-year postoperative follow-up, the HHS in the CAL group was significantly lower than that in the DM group (80.83 ± 3.91 vs. 83.17 ± 4.15, P < 0.05). The VAS score in the CAL group increased maximally at the 1-year follow-up (2.07 ± 0.91 vs. 1.49 ± 0.85, *P* < 0.05). However, at the last follow-up after surgery, there were no significant differences between the two groups in terms of HHS, VAS, and SF-36 scores. Operative time and the amount of bleeding in the DM group were significantly lower than those in the CAL group (105.30 ± 29.68 vs. 94.85 ± 31.07; 355.11 ± 123.95 vs. 302.22 ± 107.68, *P* < 0.05) (Table 2). The average length of hospital stay for patients in the CAL group was 7.23 ± 3.41 days, while in the DM group, it was 7.05 ± 3.19 days. There was no significant difference between the two groups (*p* = 0.855) (Table 2). At the last follow-up, hip flexion and extension ROM were measured, and the hip range of motion in the CAL group was significantly worse than that in the DM group (69.06 ± 14.18 vs. 72.94 ± 13.19; 8.98 ± 5.48 vs. 11.59 ± 6.34, *P* < 0.05) (Table 3).

**Table 2:** Comparison of postoperative results between the two groups.

	CAL group (*n* = 96)	DM group (*n* = 103)	*Z*	*P*
HHS score				
1 year follow up	80.83 ± 3.91	83.15 ± 4.17	3.507	<0.001
Last follow up	85.65 ± 3.74	86.06 ± 2.84	0.819	0.413
VAS				
Preoperation	5.48 ± 0.69	5.46 ± 0.96	−0.277	0.782
1 year follow up	2.07 ± 0.91	1.49 ± 0.85	−4.418	<0.001
Last follow up	1.61 ± 0.84	1.65 ± 0.68	0.408	0.683
SF-36				
Preoperation	50.24 ± 7.95	51.53 ± 8.43	1.090	0.276
1 year follow up	85.21 ± 3.88	85.52 ± 3.75	0.449	0.654
Last follow up	85.43 ± 3.51	86.18 ± 3.14	1.540	0.124
Operative Time, *n* (min)	105.30 ± 29.68	94.85 ± 31.07	−2.887	0.004
Amount of bleeding (ml)	355.11 ± 123.95	302.22 ± 107.68	−3.062	0.002
Hospital stay (day)	7.23 ± 3.41	7.05 ± 3.19	0.983	0.855

Data are expressed as mean ± standard deviation. Mann–Whitney *U*-test.

SF-36 Health Questionnaire patients scores.

**Table 3:** Range of motion with patients underwent hip arthroplasty for two groups.

	CAL group (*n* = 96)	DM group (*n* = 103)	*Z*	*P*
Flexion (°) at last follow up	69.06 ± 14.18	72.94 ± 13.19	2.203	0.028
Extension (°) at last follow up	8.98 ± 5.48	11.59 ± 6.34	2.736	0.006

Data are expressed as mean ± standard deviation. Mann–Whitney *U*-test.

### Radiological measurements

In this study, it is roughly assumed that the offset of the affected limb on the patient's preoperative side is consistent with that of the healthy limb on the unaffected side. Therefore, the offset of the healthy limb measured preoperatively is assumed to be the same as the offset of the patient's affected limb before the fracture. This study includes a comparison of preoperative offsets between two groups of patients, and the results show no differences. Similarly, there is no statistically significant difference in the postoperative offset values between the two patient groups in this study.

The measurement of lower limb length for all patients was conducted in a non-weight-bearing position. During the acquisition of the anteroposterior pelvic x-ray, both hip joints of the patients were maintained at 0 degrees of extension and 15 degrees of internal rotation. At this point, the vertical distance between the bilateral landmarks of the ischial tuberosities and the lesser trochanters was measured on the x-ray. The difference between these measurements is considered as the patient's bilateral lower limb length discrepancy.

In the CAL group, the mean postoperative offset was 43.97 ± 7.90 mm, whereas in the DM group, the mean offset was 45.57 ± 7.07 mm. However, there was no significant difference between the two groups at the last follow-up. Similarly, the mean leg length discrepancy between the two groups also showed no significant difference. (Table 4).

**Table 4:** Radiological outcomes in patients who underwent total hip arthroplasty for two groups.

	CAL group (*n* = 96)	DM group (*n* = 103)	*Z*	*P*
Offset (mm)	43.97 ± 7.90	45.57 ± 7.07	1.232	0.218
Leg length discrepancy (mm)	3.07 ± 2.36	3.12 ± 3.04	−0.701	0.483
Engh score (Last follow up)	23.89 ± 2.73	25.32 ± 1.58	3.842	<0.001

Data are expressed as mean ± standard deviation. Mann–Whitney *U*-test.

### Postoperative complication

**Figure 1:**
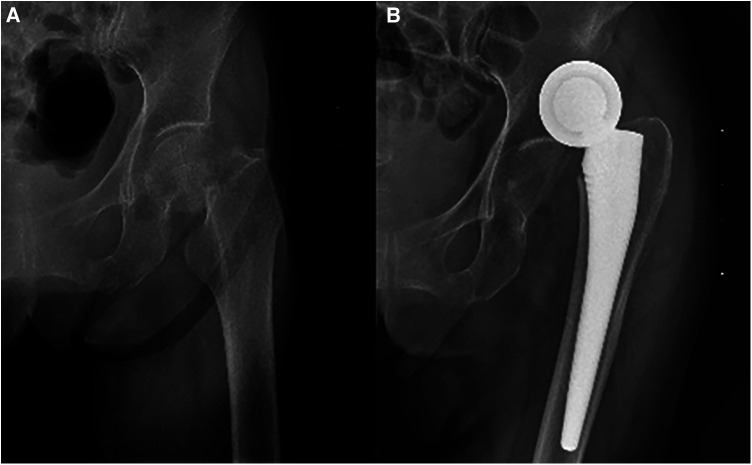
A 92-years-old female patient was diagnosed with left side femoral neck fracture. The patient underwent hemiarthroplasty. (**A**) Preoperative anteroposterior view. (**B**) Postoperative dislocation view. The patient used dual mobility with four weeks of follow-ups.

**Figure 2:**
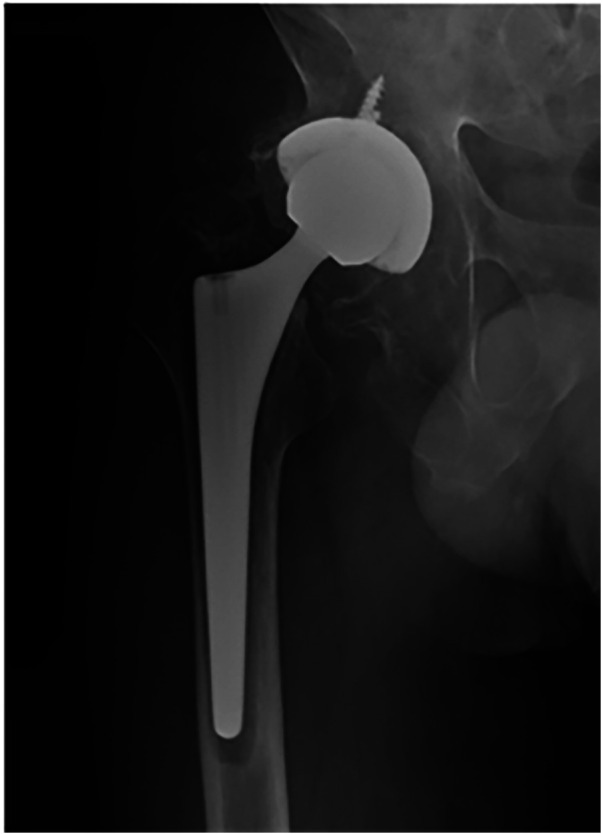
A 65-years-old male patient was diagnosed with right side femoral neck fracture. The patient underwent total hip arthroplasty. Postoperative anteroposterior view. The patient used constrained liner with five years of follow-ups.

Periprosthetic femoral fractures occurred in 2 patients in each group. The incidence of dislocation was higher in the DM group (Figure 1). However, the aseptic loosening incidence was higher in the CAL group (Figure 2). Table 5 presents the last follow-up outcomes of the two groups.

**Table 5:** Incidence of complications in two groups of patients.

Patients’ characteristics	CAL group (*n* = 96)	DM group (*n* = 103)	*t*	*P*
Periprosthetic femoral fracture				
Yes	2	2	0.005	0.943^a^
No	94	101		
Dislocation				
Yes	1	9	6.167	0.013^a^
No	95	94		
Aseptic loosening				
Yes	8	2	5.257	0.022^a^
No	88	101		
Infect				
Yes	2	1	0.414	0.520^a^
No	94	102		

^a^ Chi-square test.

All four patients experienced intraoperative periprosthetic fractures, all of which occurred in the proximal femur and were classified as stable A1-type fractures. Intraoperatively, implant stability was confirmed, obviating the need for additional fixation and allowing for early weight-bearing.

The infection rate in the CAL group was 2.1% (2 out of 94 cases), occurring within 5 days postoperatively. In the DM group, the infection rate was 1% (1 out of 102 cases), observed on the 6th day post-surgery. In both cases, the infections manifested as wound exudation. Patients were treated with intravenous cefazolin sodium at 1 g twice daily, and the treatment continued for 3 days after the disappearance of exudation. Wound healing progressed well, and no secondary surgeries were required.

Within the CAL group, aseptic loosening was observed, with 6 cases involving the acetabular cup and 2 cases involving the femoral stem. These cases occurred between 3 and 5 years postoperatively. In the DM group, all implant loosening occurred on the femoral stem and happened during the 4–6-year follow-up. Loosening had a detrimental impact on patient pain and lower limb function, resulting in a reduction in the HHS. Ultimately, all patients required revision surgery.

## Discussion

Hip arthroplasty remains one of the most common methods for treating femoral neck fractures in elderly individuals. However, for patients with poor limb muscle strength, especially those with abductor deficiency, recurrent dislocation is the most common complication, further elevating the risk of revision surgery (20). To the best of our knowledge, there is a lack of available data in the literature regarding dislocation rates in elderly stroke patients with femoral neck fractures treated with DM and CLA. frequently experience reduced muscle strength in their lower limbs, which can potentially lead to early dislocations. To increase the stability of the prosthesis after surgery, implant selection is necessary to reduce the risk of further instability and failure (21–23).

In the current study, it was observed that at the one-year postoperative mark, the HHS exhibited higher values in the DM group as compared to the CAL group. Similarly, the VAS score indicated higher values in the CAL group in contrast to the DM group. However, no substantial distinction between the two groups was identified during the latest follow-up evaluation. Patients in the DM group did not require acetabular preparation during the surgical procedure, leading to a significant reduction in both operative time and blood loss in comparison to patients in the CAL group. This contributed to the comparability of VAS and HHS between the two groups in the early time, specifically within the first year. However, at the final follow-up, there were no differences observed between the two groups in terms of the HHS and VAS. This phenomenon could be attributed to the patients' prolonged engagement in rehabilitation training, which facilitated a favorable recovery of hip joint function. As a consequence, the initial discrepancies between the two groups gradually diminished. Similar findings have been corroborated by existing literature (24).

Furthermore, a noticeable distinction was evident between the VAS score and HHS in the two groups during the first year after surgery, and this distinction seemed to exhibit synchronization. Indeed, the relationship persists in which changes in VAS scores can influence the fluctuations in HHS. This correlation between the two indicators appears to remain consistent even during the final follow-up evaluation. It has shown no significant difference in postoperative SF-36 scores between the two groups at any time in this study. Some studies (25–27) have reported that patients who underwent procedures had superior SF-36 scores within the first year postoperatively compared to THA, showing a difference from the findings of this study. This inconsistency may arise from the fact that the CLA patient group in this study received a constrained liner, leading to lower early dislocation rates in that group. Consequently, patients in this group had higher SF-36 scores compared to THA patients without constrained liner, thereby resulting in a lack of comparability in SF-36 scores between the two groups at any time point. Besides physical function and body pain, this score includes various supervisor evaluation indicators, such as social function and mental health, which may be the reason for no differences between the two groups.

In our study, there was a difference in the range of motion between the two groups during the last follow-up, with the CAL group exhibiting worse joint mobility. The distinctive feature of CAL is that the polyethylene pad extends beyond the middle of the femoral head. This necessitates the surgeon to “clamp” the head into the liner during intraoperative reduction. Due to the design characteristics of CAL, joint movement is restricted at certain angles. While constrained acetabular components can enhance stability (22), they can suffer from increased stress at the bone-implant interface (28). This increased stress can potentially lead to loosening and dislocation through various mechanisms, as well as a reduced range of motion “In the CAL group, the acetabular preparation required longer surgical time, resulted in greater blood loss, and caused more damage to the surrounding tissues of the hip joint during surgery. These factors may have an impact on joint mobility.

We also evaluated the radiological results, and no significant difference in offset and leg-length discrepancy was observed. However, there was a significant difference in the Engh score, with a significant increase in the CAL group. We believe that this difference is possibly due to the design of CAL, which can result in potential impact and increased interfacial stress. These factors may elevate the risk of wear, osteolysis, and loosening stress (29). Additionally, CAL is made from Ultra-high-molecular-weight polyethylene (UHMWPE), which is known for wear and particle-induced osteolysis, contributing to prosthesis loosening and the need for prosthesis revision (30, 31).

The current study found no significant difference in the incidence of periprosthetic femoral fractures between the two groups. However, there were significant differences in dislocation and aseptic loosening. The dislocation rate was significantly higher in the DM group. In a retrospective study by Kung and Ries, the use of constrained liners in patients with abductor deficiency was found to be protective against instability (32). Recent research has also suggested that larger head sizes were beneficial for preventing recurrent instability, while constrained liners were more suitable for patients with abductor deficiency (32). On the other hand, in the CLA group, a highly cross-linked polyethylene liner was used as the prosthetic component instead of the traditional polyethylene liner. Nonetheless, this study found that in the CLA group, patients experienced more cases of prosthesis loosening due to particle disease caused by liner wear compared to the DM group. This could be attributed to two factors. Firstly, the restrictive nature of the liner in the CLA group confines the movement of the femoral head prosthesis within the acetabular liner. When patients engage in weight-bearing activities in specific positions, stress is concentrated and localized on certain areas of the liner, leading to aggravated wear in those regions. The resulting substantial generation of polyethylene debris is likely a primary cause for the occurrence of particle disease and aseptic loosening in patients. While the design of constrained liners offers protection against femoral head dislocation, it limits the range of motion, increasing the risk of impingement and aseptic loosening (29). Furthermore, the constrained liner prostheses have limited mobility compared to DM, leading to stress concentration in the acetabular lining. The wear of polyethylene lining and particle-induced effects obstruct bone integration between the prosthesis and the medial bone cortex, resulting in prosthesis loosening. Previous studies have shown that prosthesis failure can occur through various mechanisms, with long-term failure rates reported up to 42.1% (33, 34). Secondly, for patients with a dual-mobility structure (DM group), the liner wear is not confined to a single area during weight-bearing movements. As patients' lower limbs move extensively, the position of the cup-liner integral structure changes, with less pronounced concentration of stress. This reduces the production of polyethylene debris, thereby lowering the risk of particle disease and aseptic loosening. Overall, despite the utilization of a highly cross-linked polyethylene liner in the CLA group, the unique properties of the liner design and its interaction with patient movement patterns can lead to increased wear and associated complications, as observed in the study's findings.

Constrained liner designs have been associated with high failure rates, including distortion and breakage of the liner rim due to load transfer via the femoral stem (29). These findings align with our conclusions.

Additionally, since CAL is made of polyethylene material, long-term wear of polyethylene has been reported to trigger a cascade of macrophage cytokines, potentially causing osteoclastic bone resorption, osteolysis, and aseptic loosening (30, 31).

Patients in both the CAL and DM groups who experienced loosening and recurrent dislocation required revision surgery, with revision rates of 8.3% (8/96) in the CAL group and 6.9% (7/101) in the DM group.

The average length of hospital stay for patients in the CAL group was 7.23 ± 3.41 days, while in the DM group, it was 7.05 ± 3.19 days. There was no significant difference between the two groups (*p* = 0.855). In this study, there was no significant difference in the incidence of complications between the two groups (*p* = 0.992), which may explain the lack of a significant difference in hospital stay between the two groups. These results are consistent with previous researches (25, 35, 36).

Uncemented femoral stem was used in this research. Previous article reported that the risk of stem revision for aseptic loosening was lower for the uncemented stems than for the cemented stems in the 6 years follow-up (37). In addition, the cemented stem requires longer duration of the operation. which may increase the risk of infection. Furthermore, there have been studies reporting cases of severe cement reactions during the cemented prosthesis surgery, resulting in patient fatalities (38, 39). Lastly, despite the favorable initial stability of cemented hip joint prostheses, the occurrence of prosthesis loosening poses significant challenges for revision surgery. For instance, in cases of substantial femoral bone loss, a complex femoral proximal replacement procedure may be required, underscoring the considerable complexities associated with addressing cemented prosthesis failures (40).

The research has certain limitations. Since this is a retrospective single-center study, some risk factors may have been overlooked, and the sample size is limited. Additionally, the follow-up period in this study was less than 6 years, and the long-term potential impact of prostheses on bone remodeling remains unclear. Further research is necessary to investigate the correlation between clinical and imaging results and bone remodeling.

## Conclusion

In summary, we analyzed the CAL and DM groups' clinical, imaging, and postoperative complications. The results showed that both groups' HHS, VAS, and SF-36 had significant improvements compared to preoperative conditions. In addition, there was a noticeable difference in HHS and VAS between the two groups at 1-year follow-up, but this difference disappeared after more than 5 years. In terms of imaging, there are differences in Engh's score. Besides, among postoperative complications, the aseptic loosening rate of the CAL group is significantly higher than the DM group. However, the dislocation rate significantly increased in the DM group. Therefore, surgeons should pay more attention to the placement depth and angle of the prosthesis during the surgery process to improve the prognosis.

DM prognosis is more beneficial for early patient recovery, but it carries a higher risk of recurrent dislocation. CAL provides excellent stability for primary THA in high-risk patients, but attention should be given to avoiding aseptic loosening.

## Data Availability

The original contributions presented in the study are included in the article/Supplementary Material, further inquiries can be directed to the corresponding author.
